# Iodized Garlic Extract Regulates Stress Responses in Epidermal Keratinocytes via the Transcription Factor Nrf2

**DOI:** 10.1111/jocd.71228

**Published:** 2026-11-02

**Authors:** Yuki Miyauchi, Natsumi Iwasaki, Liangjing Xie, Satoshi Yano, Taichi Hara

**Affiliations:** ^1^ Laboratory of Food and Life Science, Faculty of Human Sciences Waseda University Tokorozawa Saitama Japan; ^2^ Kindai Kagaku Co., Ltd Ebina Kanagawa Japan

**Keywords:** AHR, dehydrogenase activity, HaCaT, iodized garlic extract, NAD(P)H, Nrf2, stress response

## Abstract

**Objective:**

Garlic exerts protective effects against skin stress, including oxidative damage and trauma. Iodized garlic extract (IGEx) is a cosmetic ingredient developed through iodination of garlic odor components to achieve odor reduction. Although IGEx is expected to exhibit skin stress protection properties similar to garlic, the molecular mechanisms underlying the effects of IGEx on skin cells remain unclear. Therefore, this study comprehensively analyzed the effects of IGEx on human epidermal keratinocytes (HaCaT cells), with a particular focus on the stress response mechanisms.

**Methods:**

Transcriptome analysis was conducted on HaCaT cells treated with IGEx. Nrf2 protein expression was assessed by western blotting, whereas target gene expression was evaluated by qRT‐PCR. Dehydrogenase activity was measured using Cell Counting Kit‐8. Functional analyses included hydrogen peroxide‐induced oxidative stress testing and in vitro wound healing assays, and pathway specificity was validated using Nrf2 knockout cells.

**Results:**

IGEx increased Nrf2 protein expression 1.62‐fold and induced the expression of downstream target genes, including *HMOX1*, *AKR1C1*, and *AKR1C3*. Additionally, intracellular NAD(P)H‐dependent dehydrogenase activity was enhanced in an Nrf2‐dependent manner. Furthermore, IGEx demonstrated protective effects against hydrogen peroxide toxicity and promoted wound healing.

**Conclusion:**

IGEx activated Nrf2 and regulated stress responses in HaCaT cells. The control of intracellular NAD(P)H‐dependent dehydrogenase activity has been suggested to contribute to these effects. Additionally, the involvement of *AHR* was indicated, suggesting the coordinated regulation of Nrf2 in stress responses. This study provides a molecular foundation for the potential applications of IGEx as a cosmetic ingredient.

## 1 Introduction

Human skin is perpetually subjected to a range of environmental stressors, such as ultraviolet (UV) radiation, harmful chemicals, and physical damage, all of which contribute to the degradation of skin barrier function [1, 2, 3]. In response to these stressors, skin cells activate various defense mechanisms, notably antioxidant and xenobiotic responses mediated by the transcription factor Nrf2 (nuclear factor erythroid 2‐related factor 2; *NFE2L2*), which are of paramount importance. Nrf2 regulates the transcription of various antioxidant and xenobiotic‐metabolizing enzymes, including heme oxygenase‐1 (HMOX1) [4, 5] and the aldo‐keto reductases family (AKR1Cs) [6]. The essential role of Nrf2 in maintaining skin homeostasis under stress conditions is demonstrated by studies indicating that Nrf2‐deficient cells exhibit significantly reduced UV protection capacity, resulting in decreased skin flexibility and epidermal hyperplasia [7].

Recent studies have indicated that plant‐derived compounds can enhance cellular stress protection by activating Nrf2 [8, 9, 10, 11, 12, 13]. The application of these plant‐based components has garnered attention as an innovative skincare strategy to combat environmental stress.

Although Nrf2 plays a crucial role in cellular defense by regulating the transcription of antioxidant and xenobiotic‐metabolizing enzymes, the functional efficacy of these enzymes is contingent upon an adequate supply of NAD(P)H, which serves as a cofactor and an electron donor [14, 15]. Notably, AKR1Cs, which are target genes of Nrf2, directly utilize NAD(P)H as a cofactor in their reduction reactions [16]. In the case of HMOX1, the electrons required for catalytic reactions are predominantly supplied by NAD(P)H through electron transport systems, such as cytochrome P450 reductase, thereby sustaining enzymatic activity [17]. To satisfy the demand for NAD(P)H, Nrf2 induces the transcription of various NAD(P)H‐generating enzymes. These enzymes include glucose‐6‐phosphate dehydrogenase (G6PD) and 6‐phosphogluconolactonase (PGD) from the pentose phosphate pathway, as well as malic enzyme 1 (ME1) and isocitrate dehydrogenase 1 (IDH1), which are associated with the TCA cycle [14, 15]. By regulating the expression of these dehydrogenases, Nrf2 ensures an adequate intracellular supply of NAD(P)H. Moreover, Nrf2 has been implicated in several energy metabolic pathways, including lipid metabolism, glycolysis, and mitochondrial metabolism [18]. Within these pathways, NAD(P)H is produced as a byproduct of the energy metabolism. However, the extent to which NAD(P)H derived from these pathways contributes to the function of Nrf2‐regulated antioxidant and xenobiotic‐metabolizing enzymes, as cofactors or electron donors, remains unclear.

As such, Nrf2 is integral to the cellular stress response by regulating the expression of antioxidant and xenobiotic‐metabolizing enzymes, as well as NAD(P)H supply, and skin defense mechanisms are not exclusively reliant on the Nrf2 pathway. Another significant pathway involves Phase I and II xenobiotic‐metabolizing processes mediated by the transcription factor aryl hydrocarbon receptor (AHR). AHR responds to exogenous toxins, such as polycyclic aromatic hydrocarbons, and induces the expression of Phase I xenobiotic‐metabolizing enzymes, such as cytochrome P450 family 1 subfamily A member 1 (CYP1A1) [19], and Phase II xenobiotic‐metabolizing enzymes, such as UDP glucuronosyltransferase family 1 member A6 (UGT1A6) [20], thereby reducing cellular toxicity.

Previously, the antioxidant response pathway driven by Nrf2 and the xenobiotic‐metabolizing pathway driven by AHR were considered to be independent pathways with no mutual relationship [21]. However, recent research has begun to reveal the existence of coordinated regulation between AHR and Nrf2. For example, the prototypical AHR agonist 2,3,7,8‐tetrachlorodibenzo‐p‐dioxin (TCDD) regulates Nrf2 transcription through AHR activation [21]. However, knowledge of this cooperative action is insufficient, leaving many aspects of the mechanism unexplored. Various plant‐derived compounds, including polyphenols, have been reported to activate both Nrf2 [8, 9, 10, 11, 12, 13] and AHR [22, 23, 24]. However, the effects of the coordinated regulation of AHR and Nrf2 on antioxidant and xenobiotic responses remain largely unexplored and represent an important area for future research.

The iodized garlic extract examined in this study is a cosmetic ingredient prepared by iodinating garlic bulb extracts. Garlic has been used for wound healing since ancient times [25] and the promotion of wound healing through collagen synthesis, angiogenesis promotion, and antimicrobial action [26, 27]. Furthermore, diverse skin stress‐protective effects have been demonstrated, including oxidative stress defense [28], xenobiotic‐metabolizing [29, 30], anti‐inflammatory action [31, 32], and UV protection [33]. Recently, Nrf2‐mediated antioxidant and xenobiotic responses have been identified as factors contributing to these effects [34, 35].

Against this background of broad physiological activity, garlic is expected to find application as a cosmetic ingredient. However, the characteristic odor of sulfur compounds, such as allyl sulfides in garlic, is a major barrier to cosmetic applications. Therefore, previous research has developed a method to reduce odor by adding iodine to garlic extract and converting the odor components into iodine compounds [36, 37]. The iodized garlic extract used in this study was obtained via this process. Iodized garlic extract is thought to enhance the practicality of garlic as a cosmetic material, while maintaining its beneficial properties.

However, the effects of iodized garlic extract on skin cells have not yet been sufficiently validated. Therefore, in this study, we comprehensively analyzed the effects of iodized garlic extract on human epidermal keratinocytes using genome‐wide transcriptome analysis. In particular, this study examined stress defense functions based on previous knowledge that garlic‐derived components activate the Nrf2 pathway. Furthermore, this study focused on the metabolic pathway changes involved in NAD(P)H supply and coordinated regulation with AHR to elucidate the novel molecular mechanisms involved in antioxidant and xenobiotic responses. Through these analyses, this study aimed to clarify the comprehensive effects of iodized garlic extract on skin cell stress responses and the underlying molecular mechanisms.

## 2 Materials and Methods

### 2.1 Sample

Iodized garlic extract was provided by Kindai Kagaku Co. Ltd., freeze‐dried and stored at 4°C. For the experiments, freeze‐dried iodized garlic extract was dissolved in water and diluted to the appropriate concentrations.

### 2.2 Cell Culture

Human keratinocyte cell line (HaCaT) was obtained from CLI Cell Lines Service (Eppelheim, Germany). HaCaT cells were cultured in Dulbecco's Modified Eagle Medium (D‐MEM; 044‐29765; Fujifilm Wako, Osaka, Japan) supplemented with 10% fetal bovine serum (FBS; 175012; Nichirei Biosciences Inc., Tokyo, Japan) and 1% penicillin–streptomycin (PS; 168‐23191; Fujifilm Wako) at 37°C in a humidified 5% CO_2_ atmosphere.

Haploid human cell lines (HAP1) wild type (WT) and HAP1 Nrf2 knockout cell lines (HAP1‐Nrf2‐KO) were obtained from Horizon Discovery (Cambridge, United Kingdom). HAP1 and HAP1‐Nrf2‐KO cells were cultured in Iscove's Modified Dulbecco's Medium (IMDM) with L‐Glutamine, Phenol Red, HEPES and Sodium Pyruvate (098‐06465; Fujifilm Wako) supplemented with 10% FBS and 1% PS at 37°C in a humidified 5% CO_2_ atmosphere.

Human follicle dermal papilla cells (HFDPC) were cultured using the method previously reported by Miyauchi et al. [38]. HFDPC were purchased from Takara Bio Inc. (Shiga, Japan). HFDPC were cultured in Follicle Dermal Papilla Cell Growth Medium (D12086; Takara Bio Inc.) supplemented with SupplementMix (Takara Bio Inc.) as a growth factor and 1% PS at 37°C in a humidified 5% CO_2_ atmosphere. For dehydrogenase activity assays, HFDPC were cultured without SupplementMix.

The human colorectal adenocarcinoma cell line (Caco‐2) was obtained from the American Type Culture Collection (VA, USA). Caco‐2 cells were cultured in D‐MEM supplemented with 10% FBS, 1% PS, and 1% MEM non‐essential amino acids (139‐15651; Fujifilm Wako) at 37°C in a humidified 5% CO_2_ atmosphere.

### 2.3 RNA Sequencing (RNA‐Seq)

HaCaT cells were treated with 1 mg/mL iodized garlic extract for 24 h. Total RNA was isolated using the ReliaPrep RNA Cell Miniprep System (Z6012; Promega Corporation, WI, USA), following the manufacturer's instructions. cDNA library construction, RNA sequencing using a NovaSeq 6000 System, and data analysis from quality check to differentially expressed genes (DEGs) analysis were performed by Novogene (Beijing, China). DEGs were determined by comparison with the same volume of water as that of the control. DEGs were visualized using ggVolcanoR [39] with absolute log_2_ fold change > 0.5 and *p*‐value < 0.05.

### 2.4 Gene Ontology (GO) Analysis

DEGs (cutoff: absolute log_2_ fold change > 0.5 and *p*‐value < 0.05) were subjected to GO analysis to investigate their associated functions. GO analysis was performed using Metascape [40] with the following datasets: GO Molecular Function and GO Biological Processes. The enriched terms were collected and grouped into clusters based on their membership similarities. A bubble plot of the clusters was visualized in R using the ggplot2 package. A network map of enriched terms was visualized using Cytoscape software. Highly interconnected subnetworks within the protein–protein interaction network were extracted using the Molecular Complex Detection (MCODE) algorithm [41] implemented in Metascape.

### 2.5 Ingenuity Pathway Analysis (IPA)

DEGs (cutoff: absolute log_2_ fold change > 0.5 and *p*‐value < 0.05) were subjected to IPA (QIAGEN, Venlo, Netherlands) to investigate their upstream regulators, Nrf2 target genes, molecular networks, diseases and functions, molecular and cellular functions, and canonical pathways. Upstream regulators were limited to endogenous biomolecules (excluding chemical compounds), and the results were visualized using the ggplot2 package in R. Network maps were visualized using the standard IPA package, and disease and function (Fx) annotations in the “Carbohydrate Metabolism” category were overlaid onto the maps. The “Diseases and Disorders” and “Molecular and Cellular Functions” categories under the “Diseases and Functions” section in IPA were used for disease‐related and functional analysis, respectively. Canonical pathways were visualized in R using the ggplot2 package.

### 2.6 Dehydrogenase Activity Assay

The dehydrogenase activity assay was performed using the method previously reported by Miyauchi et al. [38]. Dehydrogenase activity was measured using a Cell Counting Kit‐8 (CCK8, CK04; Dojindo, Japan). The 2‐(2‐methoxy‐4‐nitrophenyl)‐3‐(4‐nitrophenyl)‐5‐(2,4‐disulfophenyl)‐2H‐tetrazolium monosodium salt (WST‐8) colorimetric reagent in CCK8 exhibits color development corresponding to the amount of NADH produced by dehydrogenase enzymes [42]. Cells (HaCaT: 5.0 × 10^3^/well, HAP1‐WT and HAP1‐Nrf2‐KO: 5.0 × 10^3^/well, HFDPC: 7.0 × 10^3^/well, Caco‐2: 1.0 × 10^4^/well) were seeded into individual wells of 96‐well plates and incubated for 24 h. The cells were treated with 0.1–1 mg/mL iodized garlic extract for 24 h (HaCaT, HAP1‐WT, HAP1‐Nrf2‐KO, Caco‐2) or 72 h (HFDPC). After removing the medium using an aspirator, the CCK8 solution was added to each well and incubated for 1 h. The intensity of orange‐colored formazan dye was determined at a wavelength of 450 nm using a Multiskan FC microplate photometer (Thermo Scientific, Waltham, MA, USA). The dehydrogenase activity was expressed as the optical density ratio of the treatment to that of the control.

### 2.7 Mitochondrial Dehydrogenase Activity

Mitochondrial dehydrogenase activity was measured using an MTT (341–01823; Dojindo). HaCaT cells (5.0 × 10^3^/well) were seeded into individual wells of 96‐well plates and incubated for 24 h. HaCaT cells were treated with 0.1–1 mg/mL iodized garlic extract for 24 h. After removing the medium using an aspirator, the MTT solution was added to each well and incubated for 2 h. After removing the MTT solution using an aspirator, DMSO was added to each well to dissolve the formazan dye. The intensity of reddish‐purple formazan dye was determined at a wavelength of 535 nm using a Nivo Multimode Plate Reader (PerkinElmer U.S. LLC, Shelton, CT, USA). The mitochondrial dehydrogenase activity was expressed as the optical density ratio of the treatment to that of the control.

### 2.8 Cell Counting Assay

HaCaT cells (3.0 × 10^4^/well) were seeded into individual wells of 24‐well plates and incubated for 24 h. HaCaT cells were treated with 1 mg/mL iodized garlic extract for 24 h. After aspirating the medium, HaCaT cells were washed twice with 1 × PBS diluted in water (10 × D‐PBS(−); 048–29805; Fujifilm Wako). Following detachment with TrypLE (12604‐021; Thermo Scientific), HaCaT cells were collected and counted using a hemocytometer. Cell number was expressed as a percentage of the control group.

### 2.9 Western Blotting (WB)

HaCaT cells were treated with 1 mg/mL iodized garlic extract for 4 h. Because Nrf2 protein stabilization precedes the transcriptional induction of its downstream target genes, and because tBHQ has been reported to increase Nrf2 protein levels in HepG2 cells from 15 min to 8 h, with a subsequent decline by 24 h [43], 4 h was selected as an informative early time point for the western blotting analysis. Whole cells were lysed in Tris‐Triton buffer containing 50 mM Tris–HCl (pH 7.4), 150 mM NaCl, 1 mM EDTA, 1% TritonX‐100, and a protease inhibitor cocktail. The cell lysates were centrifuged at 15,000×*g* for 15 min, and the supernatants were collected. The protein concentration was determined using a Protein Assay BCA Kit (297–73101; Fujifilm Wako). Equal amounts of lysate protein were run on SDS‐PAGE and electrophoretically transferred using a Trans‐Blot Turbo Transfer System (Bio‐Rad, Hercules, CA, USA) with Mini 0.2 μm PVDF transfer packs (#1704156; Bio‐Rad). The membrane was first blocked with TBST buffer (500 mM NaCl, 20 mM Tris–HCl (pH 7.4), and 0.1% Tween 20) containing 5% nonfat dry milk and then incubated with Nrf2‐specific antibodies (EP1808Y; Abcam, Cambridge, UK) overnight at 4°C and HRP‐conjugated secondary antibodies for another 1 h. Bound antibodies were detected using the ECL system, and the relative amounts of proteins associated with specific antibodies were quantified using FUSION SOLO S (Vilber Lourmat, Marne‐la‐Vallée, France).

### 2.10 Quantitative Real‐Time PCR (qRT‐PCR)

qRT‐PCR was performed using the method previously reported by Miyauchi et al. [38]. HaCaT cells were seeded into each well of six‐well plates (1.5 × 10^5^/well). HaCaT cells were treated with 1 mg/mL iodized garlic extract for 24 h. Total RNA was isolated using a ReliaPrep RNA Cell Miniprep System, following the manufacturer's instructions. Reverse transcription of RNA into cDNA was performed using ReverTra Ace qPCR RT Master Mix with gDNA Remover (FSQ‐301; TOYOBO, Osaka, Japan), according to the manufacturer's instructions. Briefly, after incubating at 65°C for 5 min, the extracted RNA was reacted with 4 × DN Master Mix at 37°C for 5 min, then reverse‐transcribed with 5 × RT Master Mix II at 37°C for 15 min, and subsequently at 98°C for 5 min. Real‐time PCR was performed using a Thermal Cycler Dice Real Time System III (TaKaRa, Shiga, Japan) with TB Green Premix Ex Taq II (Tli RNaseH Plus; TaKaRa). The thermal cycling conditions were 95°C for 30 s, followed by 40 cycles of 5 s at 95°C and 30 s at 60°C. The sequences of the primers used for the qRT‐PCR analysis are listed in Table S1. The mRNA levels were calculated using the ΔΔCt method and normalized to *GAPDH* as an internal control.

### 2.11 Cytoprotective Assay Against Hydrogen Peroxide

HaCaT cells (5.0 × 10^3^/well) were seeded into individual wells of 96‐well plates and incubated for 24 h. HaCaT cells were pretreated with 1 mg/mL of iodized garlic extract for 24 h, followed by exposure to 0–200 μM hydrogen peroxide for 24 h. After removing the medium using an aspirator, the CCK8 solution was added to each well and incubated for 1 h, and dehydrogenase activity was measured.

### 2.12 The Wound Healing Scratch Assay

The wound healing scratch assay was performed based on the method previously reported by Kgosana et al. with modifications [44]. Cells (HaCaT: 3.0 × 10^5^/well) were seeded into individual wells of 12‐well plates and incubated for 24 h to ensure the establishment of a monolayer. After removing the medium using an aspirator, a scratch across the cell monolayer was introduced across the well diameter using a 1,000 μL pipette tip. The scratch was washed three times gently with 1 mL of non‐additive medium. The cells were treated with 1 mg/mL iodized garlic extract for 20 h. In preliminary experiments, the scratched area was completely closed at 24 h in both the control and iodized garlic extract‐treated groups, which precluded accurate quantification of the difference between groups; therefore, 20 h was selected as the endpoint. After removing the iodized garlic extract using an aspirator, photomicrographs of the wounds were taken using a BZ‐X800 microscope (Keyence, Osaka, Japan), and a BZ‐X800 Analyzer (Keyence) was used to measure the surface area of the scratch. Measurements were used to calculate the percentage of wound closure using the following equation:

$$\text{Wound closure}\;\left(\%\right)=\left(\left(A_{\text{0h}}-A_{20h}\right)/A_{\text{0h}}\right)\times 100$$

where *A*₀ₕ represents the area of the wound at 0 h and *A*₂₀ₕ represents the area of the wound at 20 h.

### 2.13 Chemical Analysis

The total sugar content was measured using the phenol‐sulfuric acid method. A volume of 0.4 mL of iodized garlic extract, diluted in water, was introduced into a glass tube, followed by the addition of 0.4 mL of 5% (w/w) phenol and 2 mL of sulfuric acid. After 30 min (mixed once after 10 min), the absorbance was measured at 490 nm using a UVmini‐1240 spectrophotometer (Shimadzu, Kyoto, Japan). The total sugar content was calculated using a glucose calibration curve.

Total protein content was quantified using the bicinchoninic acid (BCA) method with the Protein Assay BCA Kit (297‐73101; Fujifilm Wako), according to the manufacturer's instructions. The protein content was calculated based on an albumin calibration curve.

The free sugar content of iodized garlic extract was determined using HPLC. Ten mg of iodized garlic extract was mixed with 1 mL of acetonitrile: water (75:25) and sonicated for 1 h. After centrifugation (6,200 rpm, 5 min), the supernatant was filtered using a 0.22 μm syringe filter. Free sugars were analyzed using HPLC LC‐2050C 3D (Shimadzu) with a Shodex Asahipak NH2P‐50 4E column (4.6 mm I.D. × 250 mm, Shoko Science Co. Ltd., Yokohama, Japan). The mobile phase was acetonitrile: water (75:25) and the flow rate was 1.0 mL/min. A refractive index (RI) detector RID‐20A (Shimadzu) was used, and the column oven temperature was set at 30°C. Rhamnose, arabinose, fructose, galactose, and glucose were used as the monosaccharide standards.

The total carbon, hydrogen, and nitrogen (CHN) contents and free amino acids were determined by the Kanagawa Institute of Industrial Science and Technology. CHN analysis was performed using a Micro Corder JM‐11 (J‐Science Lab, Kyoto, Japan), and free amino acids were analyzed by LC–MS/MS using an AccQ‐Tag derivatization system (Waters, Milford, MA, USA).

X‐ray fluorescence analysis of iodized garlic extract was performed using an X‐ray analytical microscope XGT‐5000WR (HORIBA, Kyoto, Japan).

### 2.14 Statistical Analysis

Each data point represents mean ± SEM. Statistical analyses were performed using two‐tailed Student's *t*‐test. ^†^ *p* < 0.1 was considered a significant tendency, and *p < 0.05, **p < 0.01, ***p < 0.001 were considered significant differences.

## 3 Results

### 3.1 Iodized Garlic Extract Enhances Dehydrogenase Activity in HaCaT Cells

To comprehensively analyze the effects of iodized garlic extract on skin cells, bioinformatic analysis using genome‐wide transcriptome profiling was conducted. After RNA‐Seq analysis of the transcriptome of HaCaT cells treated with 1 mg/mL iodized garlic extract for 24 h, GO analysis was performed based on information from the DEGs. Figure 1A shows the top 20 clusters ranked by *p*‐value. In Molecular Functions (MF), “Monooxygenase activity” (−log_10_ *p*‐value: 11.63, Counts: 11, Enrichment Factor: 22.70) related to substrate oxidation reactions showed the highest statistical significance, while in Biological Processes (BP), “Monocarboxylic acid metabolic process” (−log_10_ *p*‐value: 9.47, Counts: 17, Enrichment Factor: 7.07) related to lipid metabolism showed the highest statistical significance.

**FIGURE 1:**
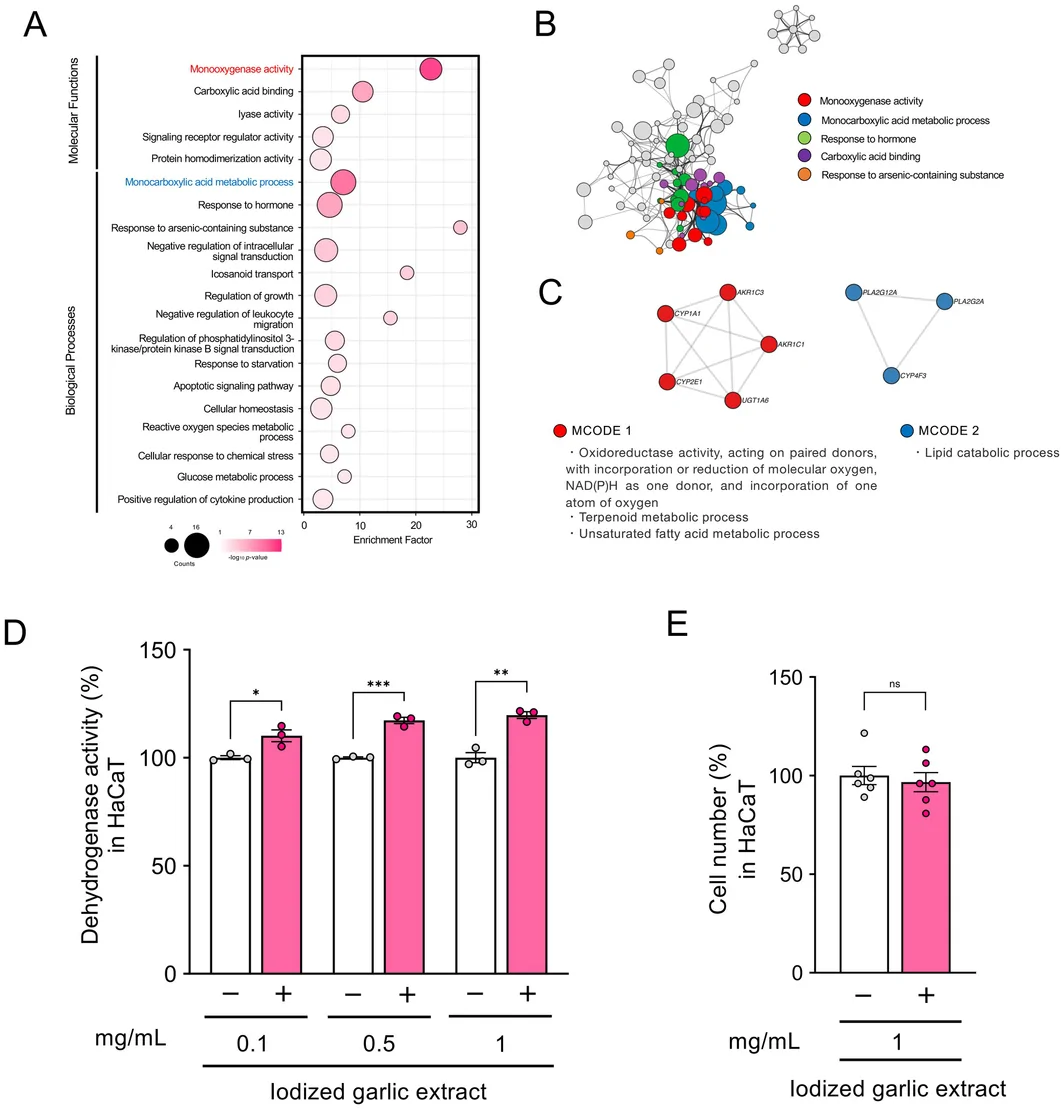
Functional analysis of iodized garlic extract based on GO analysis and in vitro validation. HaCaT cells were treated with 1 mg/mL iodized garlic extract for 24 h. Transcriptome analysis was performed by RNA‐Seq. DEGs were identified between the iodized garlic extract and control groups (*p* < 0.05, |log_2_ fold change| > 0.5). Gene Ontology (GO) analysis was performed using Metascape with the following data sets: GO Molecular Function and GO Biological Processes. Terms were collected and grouped into clusters based on their membership similarities. (A) Bubble plot of the top 20 clusters based on *p*‐values with their representative enriched terms (one per cluster). (B) Network map of enriched terms colored by cluster ID. (C) The Molecular Complex Detection (MCODE) components extracted from protein–protein interaction network. (D) Dehydrogenase activity in HaCaT cells. HaCaT cells were treated with 0.1–1 mg/mL of iodized garlic extract for 24 h, and dehydrogenase activity was measured using CCK8. (E) Cell number of HaCaT cells. HaCaT cells were treated with 1 mg/mL iodized garlic extract for 24 h, and cell number was counted under a microscope using a hemocytometer. The data represent the mean ± SEM (D, *n* = 3; E, *n* = 6). Asterisks indicate significant differences, determined using the Student's t‐test, *p < 0.05, **p < 0.01, ***p < 0.001 versus the same volume of water as a control.

Next, to infer the centers of these clusters, network analysis was performed. Only the top five clusters by *p*‐value are color‐coded, as shown in Figure 1B. “Monooxygenase activity” and “Monocarboxylic acid metabolic process,” which showed the highest statistical significance in MF and BP respectively in Figure 1A, were shown to form the center of the network. Furthermore, modules that were tightly coordinated within this network were extracted using the MCODE algorithm. MCODE is an algorithm that detects high‐density modules from protein–protein interaction networks. The modules extracted by MCODE are likely functionally important protein complexes within complex biological networks. As shown in Figure 1C, two MCODE clusters were identified. MCODE 1 comprised *AKR1C3*, *AKR1C1*, *UGT1A6*, *CYP2E1*, and *CYP1A1*, whereas MCODE 2 comprised *PLA2G12A*, *PLA2G2A*, and *CYP4F3*. These gene sets are known to participate in functions involved in cellular stress responses, including antioxidant response (*AKR1C1* and *AKR1C3*) [6], xenobiotic metabolism (*CYP1A1*, *CYP2E1*, *UGT1A6*, *AKR1C1*, and *AKR1C3*) [6, 45, 46], lipid metabolism, and regulation of inflammatory responses (*PLA2G12A*, *PLA2G2A*, and *CYP4F3*) [47, 48]. In addition, functional annotation based on enrichment analysis showed that these gene clusters were associated with “oxidoreductase activity, acting on paired donors, with incorporation or reduction of molecular oxygen, NAD(P)H as one donor, and incorporation of one atom of oxygen,” “terpenoid metabolic process,” “unsaturated fatty acid metabolic process,” and “lipid catabolic process” (Figure 1C). These findings suggest that one of the important functions of iodized garlic extract may be the modulation of NAD(P)H‐dependent redox reactions, as well as lipid and fatty acid metabolism.

To further clarify the functional significance of iodized garlic extract, we examined whether the biological functions indicated by the MCODE analysis were altered. As an initial approach, we used the CCK8 assay, a simple method that reflects both intracellular NAD(P)H levels and overall metabolic activity of the cells. The CCK8 assay is a colorimetric method based on the reduction of the water‐soluble tetrazolium salt, WST‐8, by cellular dehydrogenases. Because these dehydrogenases are involved in major energy‐producing pathways, including lipid metabolism, glycolysis, and the tricarboxylic acid (TCA) cycle, the assay provides an index of overall intracellular metabolic activity. In addition, since these dehydrogenases catalyze redox reactions using NAD(P)H as a cofactor, CCK8 is also known to reflect intracellular NAD(P)H levels [49].

As shown in Figure 1D, treatment with 0.1, 0.5, and 1 mg/mL of iodized garlic extract increased cellular dehydrogenase activity to 110%, 117%, and 120%, respectively, in HaCaT cells (*p* < 0.05, 0.001, and 0.01, respectively). Among these concentrations, 1 mg/mL produced the most robust and reproducible concentration‐dependent increase in dehydrogenase activity and was therefore adopted as the working concentration for the subsequent RNA‐Seq and mechanistic analyses; no morphological signs of cytotoxicity were observed at any concentration. Because the CCK8 (WST‐8) signal reflects the intracellular NAD(P)H‐dependent reductase capacity, the increase in signal was not interpreted as a cell‐viability readout. However, because the CCK8 assay is commonly used as an indicator of viable cell numbers [42, 50], we measured HaCaT cell numbers directly using a hemocytometer to exclude the possibility that the observed increase in signal was attributable to an increase in cell number. As shown in Figure 1E, there was no significant change in the cell number between the 1 mg/mL iodized garlic extract treatment group and the untreated group (*p* = 0.63). The increase in the CCK8 signal reflects enhanced NAD(P)H‐dependent metabolic activity rather than increased cell proliferation, as confirmed by direct cell counting (Figure 1E). Furthermore, the MTT assay showed that 0.5 and 1 mg/mL iodized garlic extract enhanced mitochondrial dehydrogenase activity in HaCaT cells (Figure S1).

These results indicate that iodized garlic extract increased intracellular dehydrogenase activity without affecting HaCaT cell number. This suggests an increase in energy metabolism levels in skin cells due to iodized garlic extract treatment. Furthermore, the observation of this effect both before and after iodination (Figure S2) suggests that the iodination process does not impair functionality.

### 3.2 Iodized Garlic Extract Enhances the Protein Expression of the Transcription Factor Nrf2

Subsequently, we investigated the molecular mechanisms by which iodized garlic extract enhanced dehydrogenase activity. First, we analyzed the major regulatory factors upstream of the DEGs using QIAGEN IPA. The top 10 regulatory factors ranked by *p*‐values are shown in Figure 2A. *NFE2L2*, the gene encoding the transcription factor Nrf2 (*z*‐score: 1.14), showed the highest statistical significance (−log_10_ *p*‐value: 6.21). Therefore, since Nrf2 activation was suspected, protein expression was examined by western blotting. In the 1 mg/mL iodized garlic extract treatment group, Nrf2 protein levels in HaCaT cells increased 1.62‐fold (*p* < 0.001) compared to those in the untreated group (Figure 2B). These results suggest that the transcription factor Nrf2 is a major regulatory factor in iodized garlic extract. Furthermore, the result that *AHR* (*z*‐score: 1.32) showed the second highest statistical significance (−log_10_ *p*‐value: 5.40) (Figure 2A) also suggests *AHR* activation.

**FIGURE 2:**
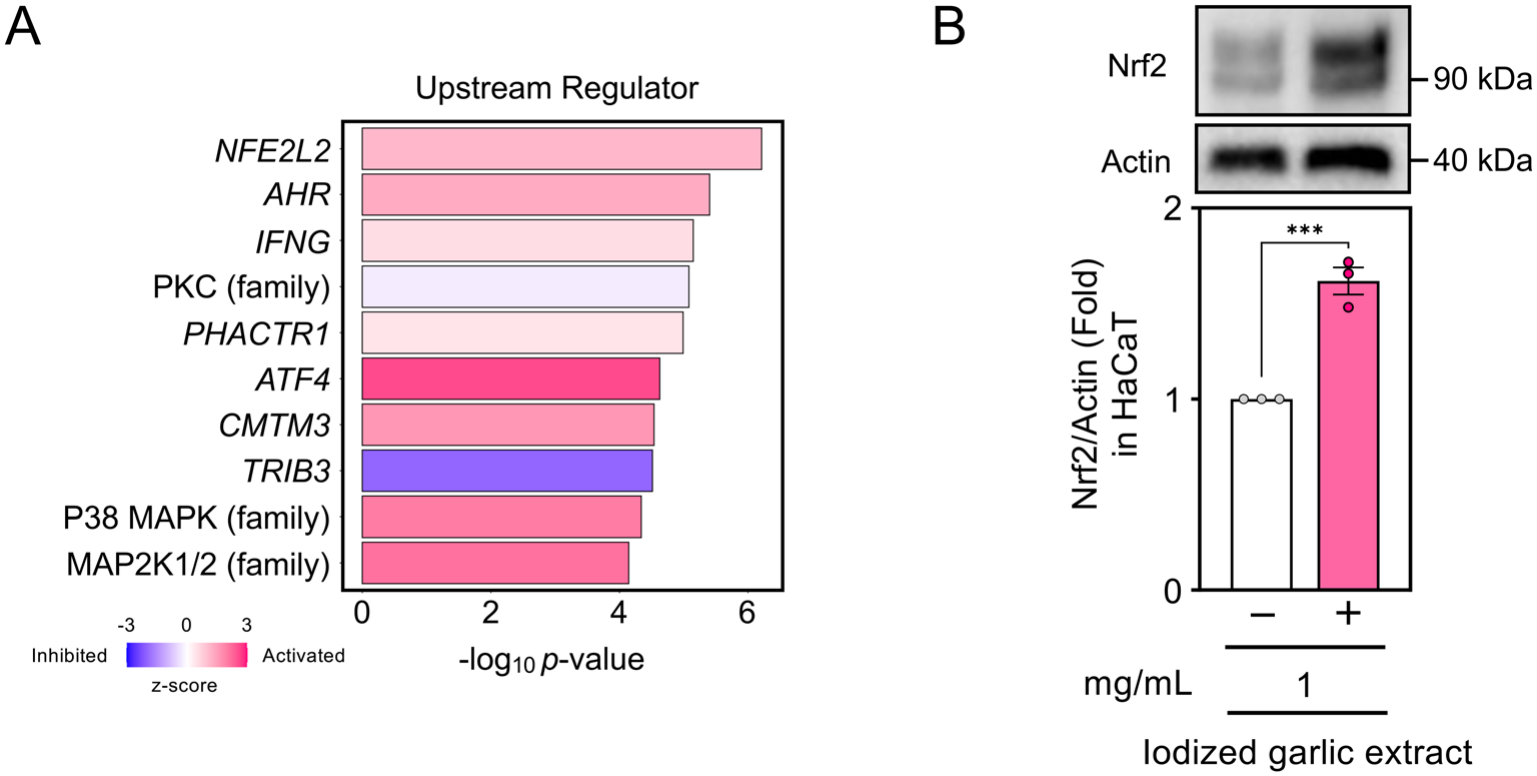
Upstream regulator analysis of DEGs by IPA and validation by western blotting. (A) Bar graph of the predicted upstream regulators. The x‐axis represents the −log_10_ *p*‐value, and the color indicates the *z*‐score. (B) Western blot analysis with specific antibodies against Nrf2 (whole cells). HaCaT cells were treated with 1 mg/mL of iodized garlic extract for 4 h, and the protein expression of Nrf2 was quantified by western blotting. The data represent the mean ± SEM (B, *n* = 3). Asterisks indicate significant differences, determined using the Student's *t*‐test, ***p < 0.001 versus the same volume of water as a control.

### 3.3 Possibility That Nrf2 Regulates Dehydrogenase Activity

Next, to verify the possibility that Nrf2 controls dehydrogenase activity, we analyzed Nrf2 target genes and their associated functions. Table 1 shows the Nrf2 target genes among DEGs. IPA identified *AKR1C1*, *AKR1C3*, *DDIT3*, *DDIT4*, *HMOX1*, *IL1RN*, *SESN2*, *SLC7A11*, *TSC22D3*, and *UGT1A6* as Nrf2 target genes. Table 2 shows the descriptions, −log_10_ *p*‐values, and genes for MCODE 1 and 2. The constituent genes of MCODE 1, which are related to oxidation–reduction and metabolism, include the Nrf2 target genes *AKR1C1*, *AKR1C3*, and *UGT1A6*.

**TABLE 1:** Analysis of Nrf2 target genes among DEGs using IPA.

Upstream regulator	Target molecules in dataset
*NFE2L2*	*AKR1C1*, *AKR1C3*, *DDIT3*, *DDIT4*, *HMOX1*, *IL1RN*, *SESN2*, *SLC7A11*, *TSC22D3*, *UGT1A6*

**TABLE 2:** Detailed characterization of MCODE clusters identified using Metascape.

MCODE	Description	−log_10_ *p*‐value	Genes
MCODE 1	Oxidoreductase activity^a^	11.2	*AKR1C1*, *AKR1C3*, *UGT1A6*, *CYP1A1*, *CYP2E1*
	Terpenoid metabolic process	9.3	
	Unsaturated fatty acid metabolic process	9.2	
MCODE 2	Lipid catabolic process	6.0	*PLA2G12A*, *PLA2G2A*, *CYP4F3*

^a^ Oxidoreductase activity, acting on paired donors, with incorporation or reduction of molecular oxygen, NAD(P)H as one donor, and incorporation of one atom of oxygen.

Subsequently, the molecular networks associated with the Nrf2 target genes were analyzed using IPA. The DEGs formed 12 molecular networks (data not shown); among these networks, those containing Nrf2 target genes are shown in Figure 3. This network is related to carbohydrate metabolism and includes four Nrf2 target genes (*AKR1C1*, *AKR1C3*, *SESN2*, and *DDIT4*). More specifically, *AKR1C1* and *AKR1C3* were associated with “Metabolism of carbohydrate,” “Metabolism of daunorubicin,” and “Metabolism of doxorubicin,” while *SESN2* was associated with “Gluconeogenesis.” Furthermore, *AKR1C1* and *AKR1C3* were shown to be directly related to two types of dehydrogenases, “3alpha‐hydroxysteroid dehydrogenase (A‐specific)” and “trans‐1,2‐dihydrobenzene‐1,2‐diol dehydrogenase,” and three types of oxidoreductases, “NADH or NADPH: quinone oxidoreductase,” “ketosteroid monooxygenase,” and “aldose reductase.” These five enzymes are all enzyme groups encoded by *AKR1C1* and *AKR1C3*. These results suggested that Nrf2 controls dehydrogenase activity by regulating the expression of its target genes.

**FIGURE 3:**
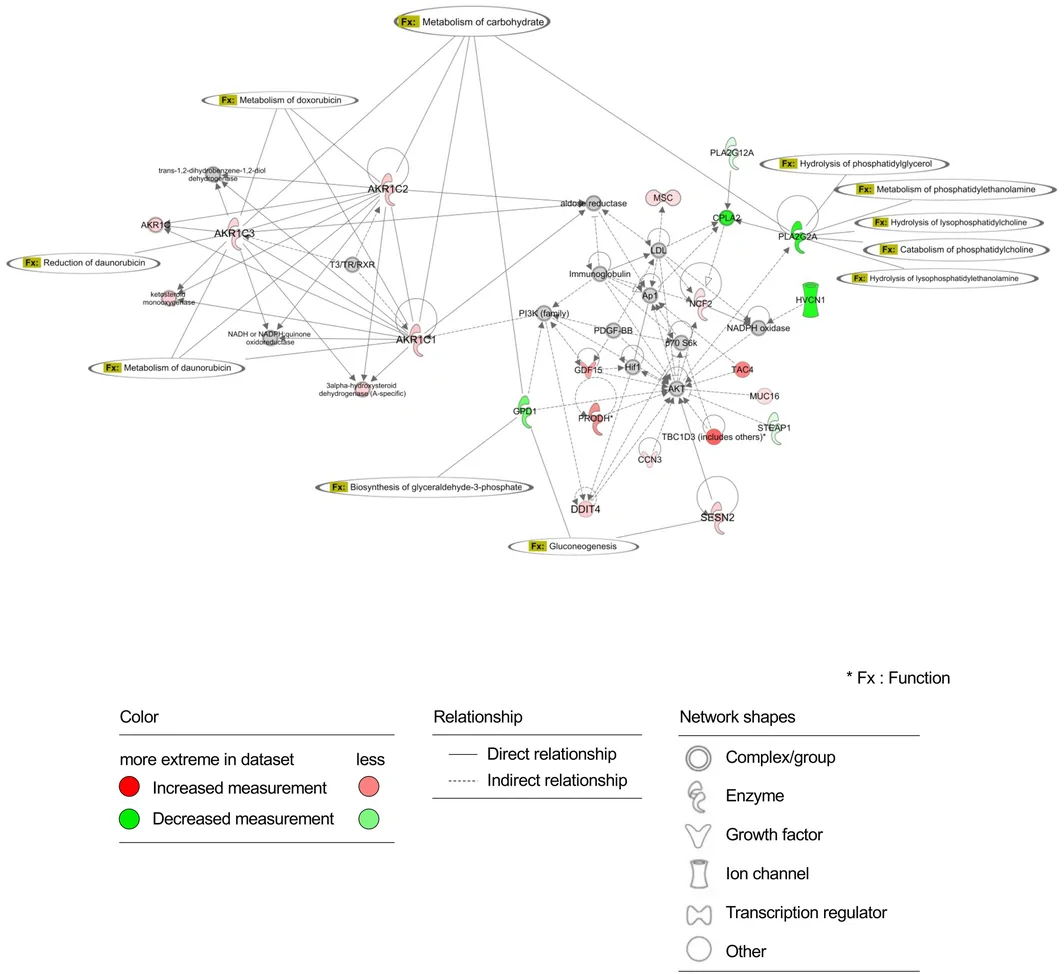
Molecular network associated with carbohydrate metabolism, including Nrf2 target genes. Network analysis was performed using IPA based on the DEGs. Network maps were visualized using the standard IPA package, and disease and function (Fx) annotations in the “Carbohydrate Metabolism” category were overlaid onto the maps.

### 3.4 Iodized Garlic Extract Enhances Dehydrogenase Activity via the Transcription Factor Nrf2

Given that bioinformatic analysis indicated a potential regulatory role of Nrf2 in dehydrogenase activity, we proceeded with validation at the cellular level. Initially, the expression of the Nrf2 target genes was verified. Figure 4A presents a comprehensive depiction of the DEGs in the form of a volcano plot. Nrf2 target genes, including *HMOX1*, *AKR1C1*, *AKR1C3*, *DDIT4*, *SESN2*, *AKR1C2*, and *CHAC1*, exhibited notable increases in expression. Subsequently, the expression of these genes was confirmed by qRT‐PCR. In the group treated with 1 mg/mL iodized garlic extract, *HMOX1*, *AKR1C1*, and *AKR1C3* demonstrated increases of 27‐fold, 4‐fold, and 7‐fold, respectively, compared to the untreated group (*p* < 0.05, 0.05, and 0.01, respectively), whereas *AKR1C2* exhibited a tendency for a 5‐fold increase (*p* < 0.1). Next, we established cells with disrupted Nrf2 genes and measured dehydrogenase activity in each cell type. HAP1 cells were used as a model system. The results are shown in Figure 5A,B. In the 0.1 and 0.5 mg/mL iodized garlic extract treatment groups, dehydrogenase activity in HAP1 wild‐type cells increased to 123% and 128%, respectively, compared to the untreated group (*p* < 0.05 and 0.01, respectively) (Figure 5A). In contrast, Nrf2 knockout cells showed no significant changes in dehydrogenase activity at any concentration (Figure 5B). These results demonstrated that iodized garlic extract enhanced dehydrogenase activity through Nrf2.

**FIGURE 4:**
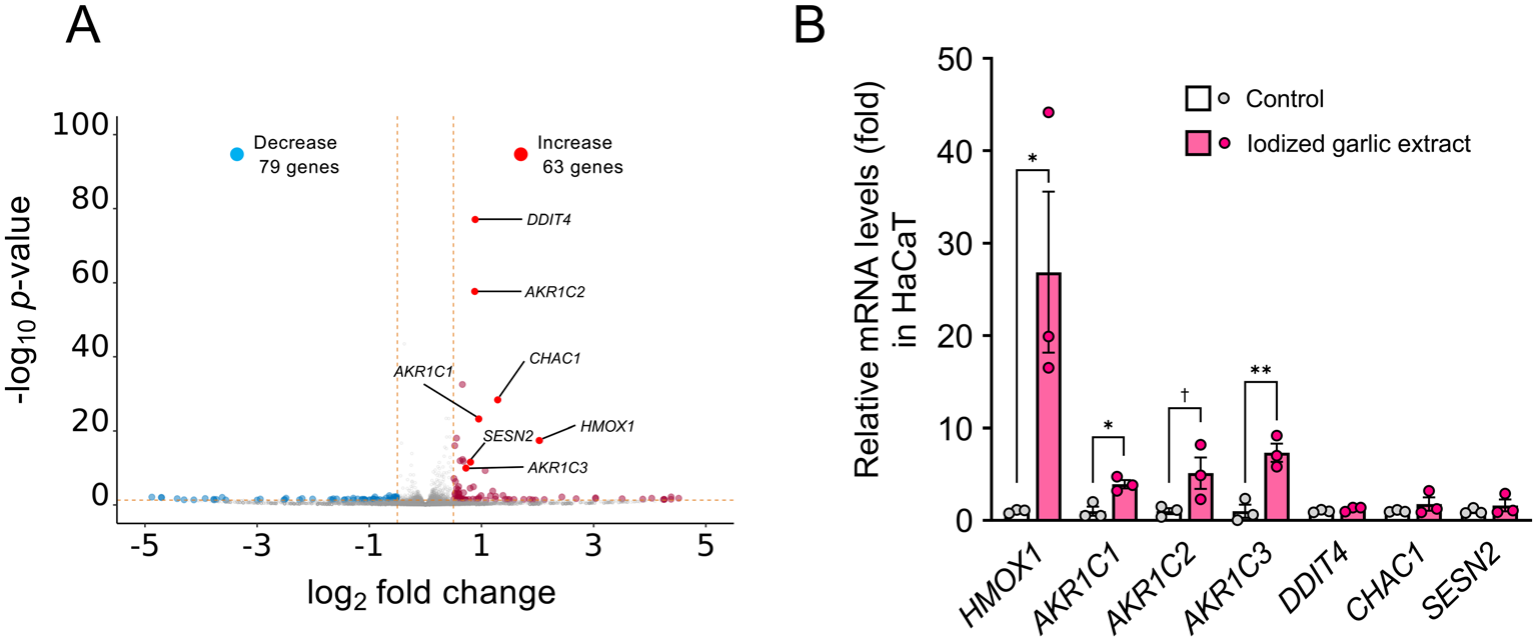
Volcano plot of DEGs with subsequent validation using qRT‐PCR. (A) Volcano plot of DEGs. DEGs were determined by comparison with the same volume of water as that of the control and by using differential expression thresholds (*p* < 0.05, and |log_2_ fold change| > 0.5). The x‐axis and y‐axis represent the log_2_ fold change and ‐log_10_ *p*‐value, respectively. The annotations pertain to genes that exhibited substantial changes in expression and demonstrated statistical significance. (B) qRT‐PCR analysis of the DEGs. After HaCaT cells were treated with 1 mg/mL of iodized garlic extract for 24 h, qRT‐PCR analysis was performed. The data represent the mean ± SEM (B, *n* = 3). Daggers and asterisks indicate significant tendencies and significant differences, respectively, determined using Student's *t*‐test, ^†^ *p* < 0.1, *p < 0.05, **p < 0.01 versus the same volume of water as a control.

**FIGURE 5:**
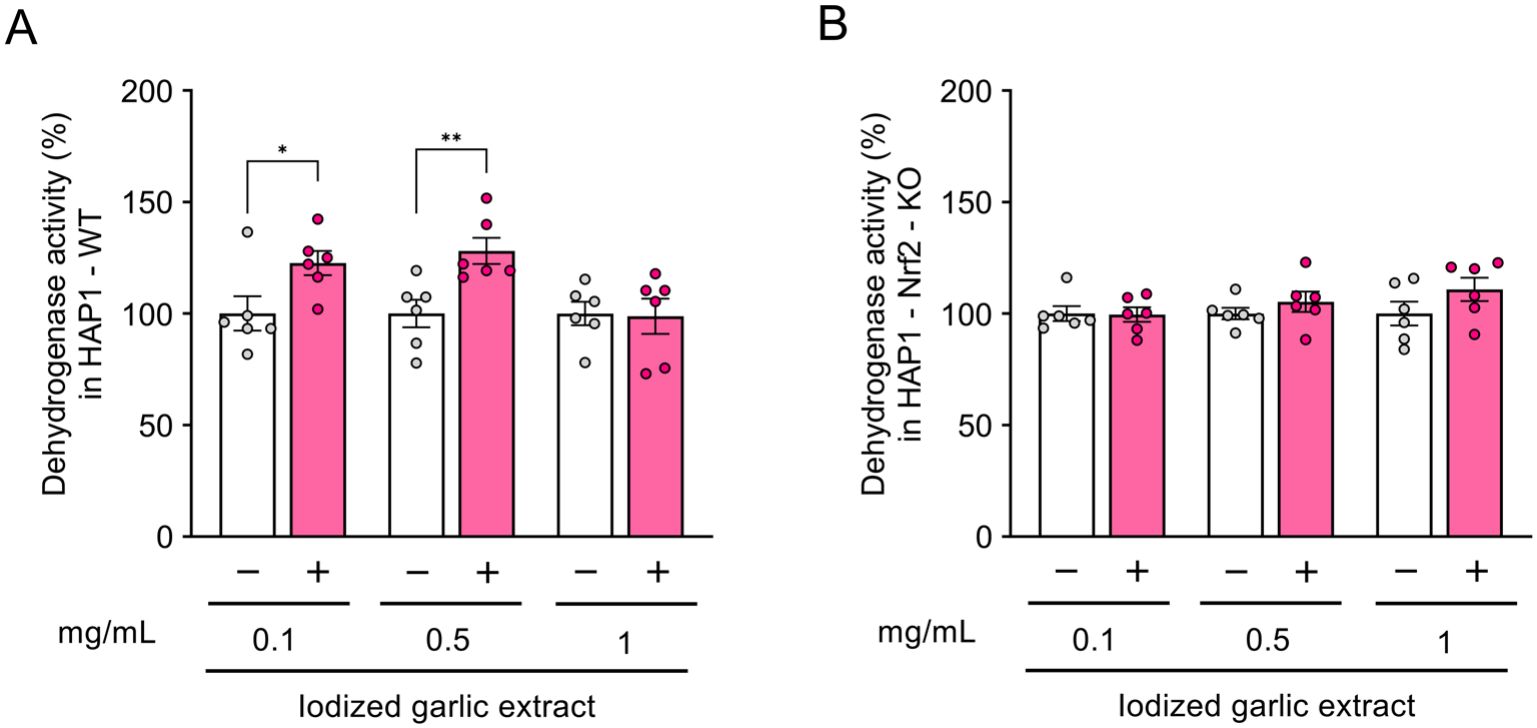
Dehydrogenase activity of (A) HAP1‐WT and (B) HAP1‐Nrf2‐KO. HAP1‐WT and HAP1‐Nrf2‐KO cells were treated with 0.1–1 mg/mL of iodized garlic extract for 24 h, and dehydrogenase activity was measured using CCK8. The data represent the mean ± SEM (*n* = 6). Asterisks indicate significant differences, determined using Student's *t*‐test, *p < 0.05, **p < 0.01 versus the same volume of water as a control.

### 3.5 Iodized Garlic Extract Protects HaCaT Cells From Hydrogen Peroxide Toxicity

We examined the applied functions of iodized garlic extract. As Nrf2 is a transcription factor that drives oxidative stress defense functions [51], the protective effect of iodized garlic extract against oxidative stress was verified. In this study, hydrogen peroxide, a well‐known oxidizing agent, was used to verify its protective effect against cellular toxicity. The results are shown in Figure 6. The vertical axis represents the relative activity of dehydrogenase enzymes related to cell survival and proliferation, and the horizontal axis shows the presence or absence of iodized garlic extract treatment and the treatment concentration of hydrogen peroxide. In the 100 and 200 μM hydrogen peroxide treatment groups, dehydrogenase activity decreased to 87% and 78%, respectively, compared to that in the untreated (0 μM) group. In contrast, in the 1 mg/mL iodized garlic extract treatment group, dehydrogenase activity recovered to 96% and 91% under 100 and 200 μM hydrogen peroxide treatment conditions, respectively (*p* < 0.01 and 0.001, respectively). These results suggest that iodized garlic extract protects against oxidative stress.

**FIGURE 6:**
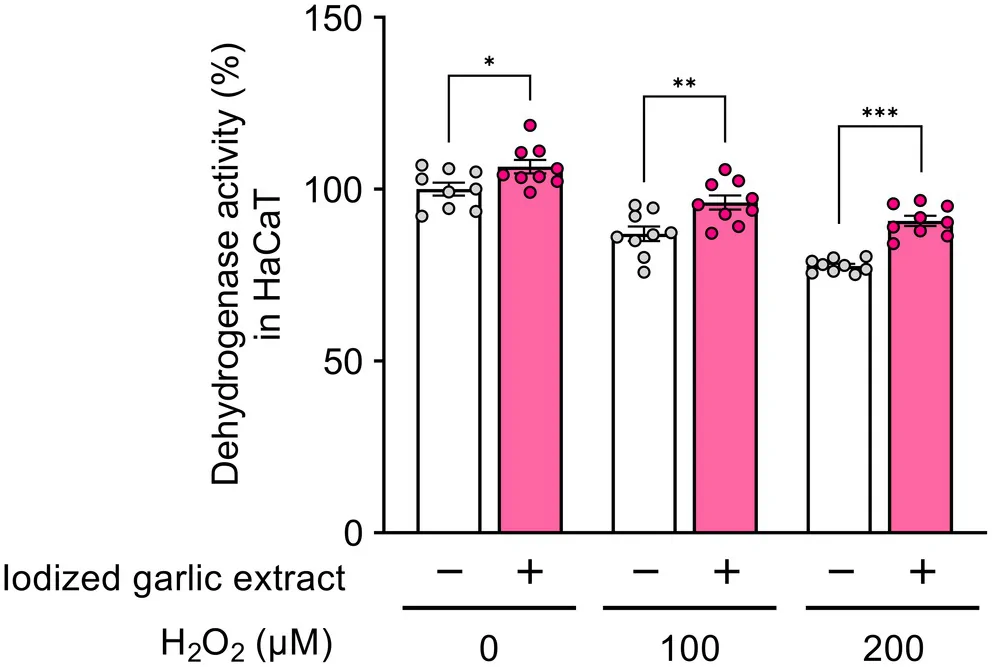
Cytoprotective effects of iodized garlic extract against hydrogen peroxide‐induced toxicity in HaCaT cells. HaCaT cells were pretreated with 1 mg/mL of iodized garlic extract for 24 h, followed by exposure to 0–200 μM hydrogen peroxide. Dehydrogenase activity was measured using CCK8. The data represent the mean ± SEM (*n* = 9). Asterisks indicate significant differences, determined using Student's *t*‐test, *p < 0.05, **p < 0.01, ***p < 0.001 versus the same volume of water as a control.

### 3.6 Iodized Garlic Extract Promotes Wound Healing Under In Vitro Conditions

We examined the effects of iodized garlic extract on diseases. First, we inferred the functionality from the DEGs information using IPA. Figure 7A shows the top 10 categories related to Disease and Disorders ranked by *p*‐value. “Organismal Injury and Abnormalities” showed the highest statistical significance (−log_10_ *p*‐value: 5.57), suggesting that iodized garlic extract exhibits functions related to damage. To verify this hypothesis, an in vitro scratch assay was performed. After culturing HaCaT cells until they formed a monolayer, we introduced pseudo‐damage using a tip and measured the closure rate after 20 h. The results are shown in Figure 7B. While the control group showed a damage closure rate of 55% after 20 h, the 1 mg/mL iodized garlic extract‐treated group showed a significant increase to 83% (*p* < 0.05). These results suggested that iodized garlic extract promotes wound healing.

**FIGURE 7:**
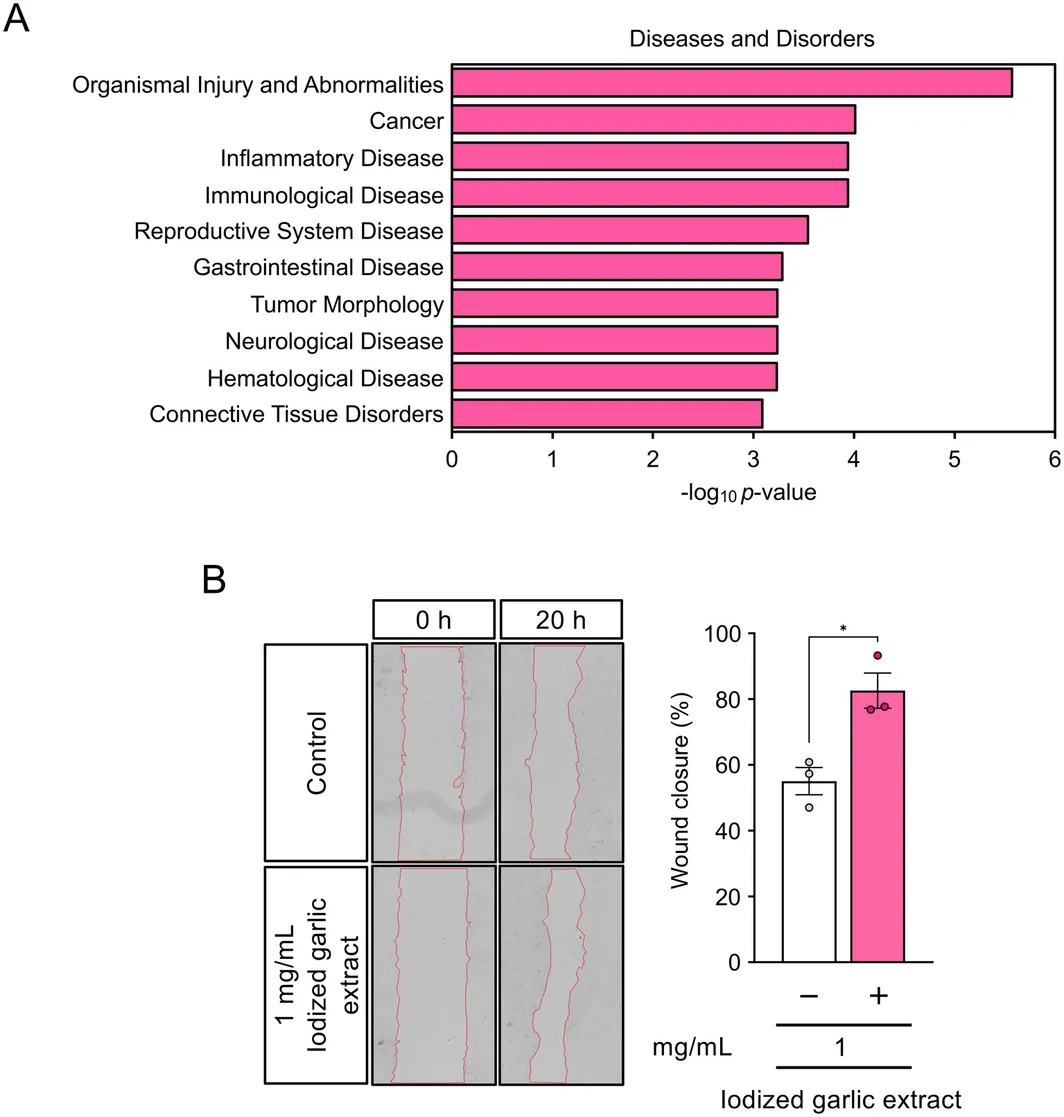
Diseases and disorders analysis via IPA and experimental validation of wound healing activity in vitro. (A) Top 10 Disease and Disorder categories based on the *p*‐values predicted by IPA. (B) Wound healing scratch assay using HaCaT cells. Microscopic images of the injured area, marked by the red boundary, are presented in the left image, whereas the rate of wound closure is depicted in the right bar graph. The data represent the mean ± SEM (B, *n* = 3). Asterisks indicate significant differences, determined using Student's *t*‐test, *p < 0.05 versus the same volume of water as a control.

## 4 Discussion

In this study, we aimed to comprehensively elucidate the effects of iodized garlic extract on skin cells through the transcriptome analysis of human epidermal keratinocytes and in vitro validation. Our results demonstrated that iodized garlic extract increased the protein expression of the transcription factor Nrf2, which drives the antioxidant and xenobiotic‐metabolizing pathways (Figure 2B). This suggests that iodized garlic extract functions in skin cell stress responses through Nrf2 activation. Indeed, iodized garlic extract showed protective effects against hydrogen peroxide toxicity (Figure 6) and wound healing effects under in vitro conditions (Figure 7B), indicating its potential contribution to maintaining skin homeostasis under stress conditions. These findings deepen our understanding of the protective mechanisms of iodized garlic extract against skin stress and provide foundational information for its application in skincare products.

However, Nrf2 activation by iodized garlic extract may exhibit dual effects, encompassing both its beneficial effects and potential risks. Nrf2 is a transcription factor that is activated in response to oxidative stress, xenobiotics, and toxins [52, 53, 54]. In this study, the fact that “Phase I‐Functionalization of compounds” showed the highest statistical significance (−log_10_ *p*‐value: 5.10, z‐score: 1.13) in canonical pathway analysis by IPA (Figure S3) suggests that iodized garlic extract may act as a toxic stimulus to skin cells. Furthermore, excessive Nrf2 activation has been shown to cause adverse effects on the skin. Sustained Nrf2 activation induces lamellar ichthyosis‐like epidermal thickening and keratinization [55]. However, treatment with 1 mg/mL of iodized garlic extract showed no significant changes in HaCaT cell numbers (Figure 1E). This result suggests that iodized garlic extract does not possess strong toxicity at this concentration.

Considering the dual nature of Nrf2, iodized garlic extract may contribute to cellular protection via hormesis‐like effects by inducing mild stress responses. A limitation of the present study is that it addressed the in vitro mechanistic effects of iodized garlic extract and did not include a formal toxicological or raw‐material safety evaluation. The iodization process underlying this ingredient was established in the 1960s [36, 37], and iodized garlic extract has since been used as a cosmetic ingredient; consistent with this use history, treatment at the concentrations tested here produced neither a reduction in cell number (Figure 1E) nor morphological signs of cytotoxicity. Nevertheless, a dedicated safety and toxicological assessment of the raw material, together with quantification of its iodine content, will be required to fully characterize the ingredient and represents an important direction for future work.

Next, we analyzed the mechanisms underlying the Nrf2‐mediated antioxidant and xenobiotic responses. Our results revealed that iodized garlic extract controlled dehydrogenase activity through Nrf2 (Figure 5). This suggests that Nrf2‐mediated antioxidant and xenobiotic responses are controlled not only by the direct transcriptional regulation of antioxidant and xenobiotic‐metabolizing enzymes, such as AKR1C1, AKR1C3, UGT1A6, and HMOX1, but also by dehydrogenase enzymes involved in NAD(P)H supply. However, well‐known Nrf2 target genes involved in NAD(P)H supply regulation, including *ME1* (log_2_ fold change: 0.17, −log_10_ *p*‐value: 1.21), *IDH1* (log_2_ fold change: 0.11, −log_10_ *p*‐value: 1.12), *G6PD* (log_2_ fold change: 0.19, −log_10_ *p*‐value: 4.31), and *PGD* (log_2_ fold change: 0.35, −log_10_ *p*‐value: 15.62) [14, 15], did not show significant expression increases. This suggests the existence of novel mechanisms for NAD(P)H supply.

On the other hand, changes in other genes potentially involved in NAD(P)H supply were confirmed. First, increased expression of proline dehydrogenase (*PRODH*), related to amino acid metabolism (log_2_ fold change: 1.91, −log_10_ *p*‐value: 1.39), was observed. PRODH has been reported to be involved in cellular protection through NAD(P)H maintenance under hydrogen peroxide stress [56]. Next, increased expression of aldolase, fructose‐bisphosphate C (*ALDOC*) (log_2_ fold change: 0.59, −log_10_ *p*‐value: 4.97), related to glycolysis, was observed. This suggests that NAD(P)H supply is accompanied by energy metabolism control through glycolysis. Indeed, analysis in the Molecular and Cellular Functions category of Diseases and Functions using IPA showed enrichment of genes related to “Energy Production” (−log_10_ *p*‐value: 5.16) (Figure S4). Furthermore, in GO Biological Processes, genes related to “Glucose metabolic process” (Counts: 4, Enrichment Factor: 7.28, −log_10_ *p*‐value: 2.64) were also enriched (Figure 1A). However, for these gene groups, there are limited reports on direct transcriptional control by Nrf2. Therefore, their contribution to NAD(P)H supply in antioxidant and xenobiotic‐metabolizing mechanisms requires further investigation.

Molecular network analysis linked *AKR1C1* and *AKR1C3* to 3alpha‐hydroxysteroid dehydrogenase activity (Figure 3). 3alpha‐Hydroxysteroid dehydrogenases are members of the AKR family and are known as steroid hormone‐metabolizing enzymes [57]. This metabolic reaction is reversible and catalyzes either substrate reduction reactions that consume NAD(P)H or substrate oxidation reactions that produce NAD(P)H, depending on the substrate and cofactors NAD(P)H and NAD(P)^+^ [57]. It should be noted, however, that in intact cells AKR1C enzymes act predominantly as NAD(P)H‐consuming ketosteroid reductases, because their NAD(P)H‐generating oxidase direction is potently inhibited by physiological NADPH concentrations [57, 58, 59]. Therefore, rather than serving as a direct source of NAD(P)H, the induction of these enzymes more likely reflects an increased NAD(P)H demand and turnover accompanying activation of Nrf2‐driven antioxidant and xenobiotic responses by iodized garlic extract. Consistent with the upregulation of *ALDOC*, the requisite NAD(P)H may be supplied through glycolysis and the pentose phosphate pathway [60]. The increase in CCK8 (WST‐8) signal is therefore not contradictory to NAD(P)H consumption by AKR1C enzymes; rather, it reflects a coordinated metabolic remodeling in which Nrf2 simultaneously upregulates NAD(P)H‐producing pathways and NAD(P)H‐consuming detoxification enzymes (AKR1C1 and AKR1C3), yielding a net increase in the cellular NAD(P)H‐dependent reductase capacity. Because the CCK8 assay reflects this net capacity rather than the absolute NAD(P)H content [61], the strong Nrf2 dependence of the response (Figure 5B) indicates that a cell‐autonomous, transcription‐factor‐driven process makes a major contribution. Direct measurement of the intracellular NAD(P)H/NAD(P) + balance will be required to clarify the precise contribution of AKR1C enzymes and other dehydrogenases to NAD(P)H homeostasis.

These results suggest that the control of antioxidant and xenobiotic responses by iodized garlic extract includes not only direct transcriptional control of antioxidant and xenobiotic‐metabolizing enzymes by Nrf2 but also coordinated regulation of metabolic pathways associated with NAD(P)H homeostasis. This study extends the understanding of Nrf2 activation mechanisms by garlic‐derived components beyond the transcriptional control of antioxidant and xenobiotic‐metabolizing enzymes, including regulation of NAD(P)H supply. However, whether NAD(P)H levels actually increase or not requires further investigation.

Furthermore, enhanced dehydrogenase activity was confirmed not only in HaCaT cells but also in HFDPC involved in hair cycle (Figure S5A) and Caco‐2, which is responsible for digestive tract barrier function (Figure S5B). Thus, control of NAD(P)H‐related metabolism by iodized garlic extract is commonly observed in cells from various tissues beyond the skin, suggesting that its stress response control mechanisms may extend to a wide range of cell types.

Thus, iodized garlic extract may induce Nrf2‐based stress responses in a wide range of cell types. Additionally, this study suggests that AHR may be involved in controlling these responses. Indeed, *AHR* showed the second‐highest statistical significance after Nrf2 in the upstream regulator analysis (Figure 2A). Furthermore, MCODE analysis to discover functionally important protein complexes from gene expression networks showed that MCODE 1 (*AKR1C1*, *AKR1C3*, *UGT1A6*, *CYP2E1*, *CYP1A1*) is composed of Nrf2 target genes *AKR1C1*, *AKR1C3*, and *UGT1A6* (Table 1) [62, 63, 64, 65, 66] and AHR target genes *CYP1A1* and *UGT1A6* (Figure 1C) [19, 20]. This suggests that the target gene groups of both pathways may functionally interact with the same molecular network, indicating the coordinated control of Nrf2 and AHR by iodized garlic extract.

Several reports exist regarding AHR activation mechanisms by plant‐derived components, particularly polyphenols, the mechanisms of which have been clarified to some extent [22, 23, 24]. For example, pelargonidin, a polyphenol, has been reported to function as an AHR ligand, inducing the expression of AHR target genes such as *CYP1A1* [67]. However, studies on AHR activation by garlic‐derived components are limited. In particular, the direct involvement of sulfur compounds in AHR and their molecular mechanisms have not been sufficiently investigated. Furthermore, cases where plant‐derived components act in coordination on both Nrf2 and AHR pathways are very limited, with only a few reports showing that coffee extract activates both Nrf2 and AHR [68]. The findings of this study suggest that iodized garlic extract facilitates antioxidant and xenobiotic responses through coordinated activation of Nrf2 and AHR. This discovery offers a novel perspective on traditional research on garlic components and significantly enhances our understanding of skin cell homeostasis. It should be noted, however, that the proposed coordinated regulation of AHR and Nrf2 in the present study is based on bioinformatic prediction using IPA upstream regulator analysis and on network module analysis using the MCODE algorithm. Direct evidence for a synergistic interaction between AHR and Nrf2—for example, functional rescue experiments using AHR antagonists or AHR‐knockout cells—awaits further investigation.

Additionally, iodized garlic extract may influence skin homeostasis through steroid hormone metabolism, beyond the stress responses suggested in this study. *AKR1C1*, *AKR1C2*, and *AKR1C3*, whose expression increased with iodized garlic extract treatment (Figure 4B), have been reported to function in the metabolism of various steroid hormones, including dihydrotestosterone, which shows strong androgenic activity, and estrogen and progesterone, which are female hormones, in addition to xenobiotic metabolism and antioxidant responses [57]. Sex hormones such as androgens and estrogens have been reported to function in keratinocyte proliferation, sebum regulation, and skin barrier function control [69].

Additionally, to determine the main active components involved in the stress response of iodized garlic extract, we examined the effects of samples before and after the iodination process on HaCaT dehydrogenase activity. Both samples showed increased HaCaT dehydrogenase activity before and after iodination (Figure S2). This result suggests that the compounds involved in dehydrogenase activity enhancement are garlic‐derived components, and that the iodination process does not impair functionality. This indicates that iodized garlic extract may contribute to the high value‐added utilization of garlic as a cosmetic ingredient by maintaining the beneficial properties of garlic while reducing its odor.

Finally, we analyzed the compositional characteristics of the iodized garlic extract. Because raw garlic is composed largely of carbohydrates (33.1 g/100 g) and proteins (6.36 g/100 g) [70], we first quantified the total sugar and protein content of the extract. The total sugar and total protein contents were 92.8 ± 3.9 w/w% and 3.2 ± 0.1 w/w%, respectively, indicating that iodized garlic extract is rich in saccharides (Table S2). Next, CHN elemental analysis showed that the extract contained 36.9, 6.3, and 1.6 w/w% carbon, hydrogen, and nitrogen, respectively (Table S3). Assuming that the remaining fraction consisted of oxygen, the elemental ratio was estimated to be approximately C:H:O:*N* = 1:2:1:0.04, which is consistent with the above finding that the extract contains abundant amounts of saccharides. Furthermore, HPLC analysis of free sugars detected fructose at 4.5 ± 0.1 w/w%, whereas rhamnose, arabinose, galactose, and glucose were not detected (Table S2). Assuming the total free sugar content is 4.5 ± 0.1 w/w%, the remaining carbohydrate fraction is likely composed largely of oligo‐ and/or polysaccharides. In addition, free amino acid analysis showed that arginine was the most abundant, at 478 mg/100 g, whereas the others were present only in small amounts (Table S4). X‐ray fluorescence analysis detected iodine, together with sulfur, phosphorus, and potassium, in iodized garlic extract (Figure S6). The detection of iodine provides qualitative evidence that iodine is present in the extract, consistent with the deodorizing iodination step, whereas the sulfur signal indicates the presence of sulfur‐containing components; further structural analysis will be required to determine whether garlic‐derived organosulfur compounds are retained after iodination. Although the present study partially clarifies the compositional profile of iodized garlic extract, the active components responsible for its biological effects have not been identified. This remains an important issue for future investigations.

Although iodized garlic extract is compositionally dominated by saccharides, this does not preclude Nrf2 activation, for three non‐mutually‐exclusive reasons. First, plant polysaccharides have been reported to activate the Keap1/Nrf2/ARE pathway in various cellular systems [71, 72]. However, the specific mechanisms of garlic‐derived polysaccharides, including the fructans that constitute the major storage polysaccharide of garlic, and their physiological relevance to keratinocytes remain to be fully elucidated. Second, because Nrf2 induction is determined by the efficiency of Keap1 cysteine modification rather than by mass abundance, the minor sulfur‐containing fraction detected here may contribute substantially; diallyl trisulfide, for example, activates Nrf2 through covalent modification of Keap1 Cys288 [73]. Third, molecular iodine and iodinated species generated during the deodorizing iodination step are themselves capable of inducing phase‐II antioxidant enzymes and engaging the Keap1/Nrf2 pathway [74, 75]. Although the total iodine content was not directly quantified in the present study, direct quantification of iodine and iodine‐containing species will be an important subject for future work. These candidate mechanisms are not mutually exclusive and may act additively.

In conclusion, functional research utilizing bioinformatic analysis has demonstrated that iodized garlic extract confers protection against stress in skin cells. Furthermore, its mechanism was suggested to involve not only transcriptional control of downstream genes involved in Nrf2‐mediated antioxidant and xenobiotic responses but also coordinated regulation of NAD(P)H‐related metabolism. Additionally, this study suggests the involvement of AHR, indicating that both Nrf2 and AHR transcription factors may coordinately control stress responses through common target gene groups and molecular networks. These findings deepen the understanding of the molecular mechanisms of action based on scientific evidence of iodized garlic extract, whose skin functionality research has been limited. Furthermore, the results of this study are useful as basic data for the application of iodized garlic extract to cosmetics and contribute to the high value‐added utilization of garlic‐derived ingredients.

## Author Contributions

Y.M., S.Y., and T.H. designed the study. Y.M., N.I., and L.X. performed experiments. Y.M. and S.Y. analyzed the data. Y.M. wrote the original draft. S.Y. and T.H. reviewed and edited the manuscript. S.Y. and T.H. supervised this study. All the authors have read and approved the final manuscript.

## Funding

This research was funded by the Kindai Kagaku Co. Ltd., Kanagawa, Japan.

## Ethics Statement

The authors have nothing to report.

## Conflicts of Interest

Y. Miyauchi is an employee of the Kindai Kagaku Co. Ltd. The sponsors had no role in the design, execution, interpretation, or writing of this study.

## Supporting information


**Figure S1:** Mitochondrial dehydrogenase activity in HaCaT cells. HaCaT cells were treated with 0.1–1 mg/mL of iodized garlic extract for 24 h, and mitochondrial dehydrogenase activity was measured using the MTT assay. The data represent the mean ± SEM (*n* = 7). Asterisks indicate significant differences, determined using the Student's *t*‐test, **p < 0.01, ***p < 0.001 versus the same volume of water as a control.
**Figure S2:** Dehydrogenase activity (A) before and (B) after iodination. HaCaT cells were treated with 0.1–1 mg/mL of garlic extract before and after iodination for 24 h, and dehydrogenase activity was measured using CCK8. The data represent the mean ± SEM (*n* = 4). Asterisks indicate significant differences, determined using Student's *t*‐test, *p < 0.05, **p < 0.01, ***p < 0.001 versus the same volume of water as a control.
**Figure S3:** Canonical pathway predicted by IPA with the calculated *z*‐score. The x‐axis represents the ‐log_10_ *p*‐value, and the color indicates the *z*‐score.
**Figure S4:** Top 10 Molecular and Cellular function categories based on *p*‐values predicted by IPA. The *x*‐axis represents the −log_10_ *p*‐value.
**Figure S5:** Dehydrogenase activities of (A) HFDPC and (B) Caco‐2. These cells were treated with 0.1–1 mg/mL of iodized garlic extract for 72 or 24 h, and dehydrogenase activity was measured using CCK8. The data represent the mean ± SEM (*n* = 3). Asterisks indicate significant differences, determined using the Student's *t*‐test, *p < 0.05, **p < 0.01, ***p < 0.001 versus the same volume of water as a control.
**Figure S6:** X‐ray fluorescence analysis of iodized garlic extract using X‐ray analytical microscope.

**Table S1:** The sequences of primers used for qRT‐PCR analysis.
**Table S2:** Total sugar, total protein, and free sugar contents of iodized garlic extract, measured by the phenol‐sulfuric acid method, a Protein Assay BCA Kit, and HPLC, respectively. The data represent the mean ± SEM (*n* = 3).
**Table S3:** CHN elemental analysis of iodized garlic extract, measured using a Micro Corder JM‐11.
**Table S4:** Free amino acids analyzed by LC–MS/MS using the AccQ‐Tag derivatization system.

## Data Availability

The data supporting the findings of this study are available from the corresponding author upon reasonable request.
