# Supplementary material for: Iodized Garlic Extract Regulates Stress Responses in Epidermal Keratinocytes via the Transcription Factor Nrf2
Source: J Cosmet Dermatol. 2026 Nov 2;25(11):e71228. doi: 10.1111/jocd.71228 (PMC13727960; doi:10.1111/jocd.71228)
Supplement: Supplementary file 1 [file JOCD-25-e71228-s001.pdf]

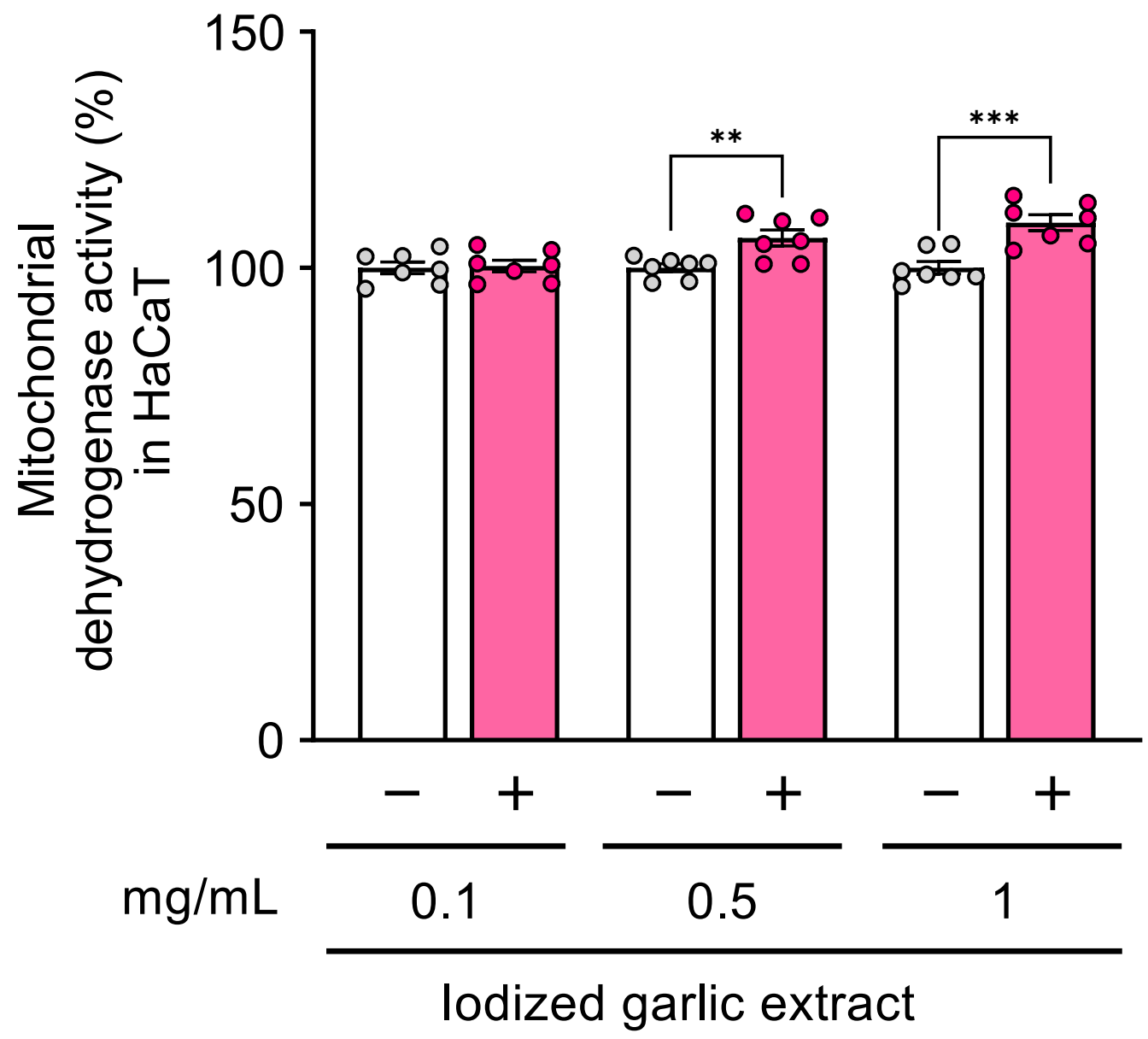

Supplementary Figure 1. Miyauchi *et al.*

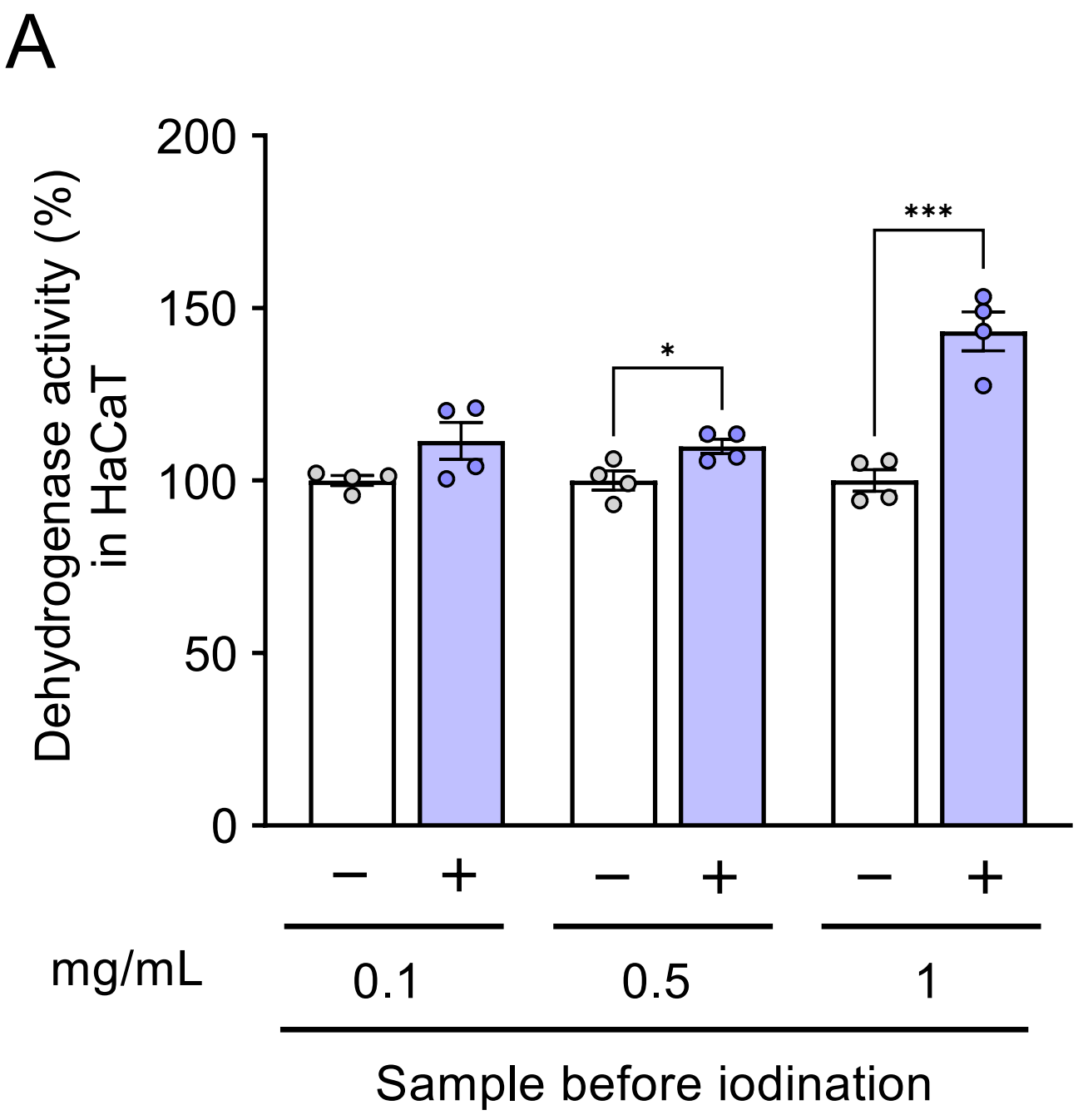

Supplementary Figure 2. Miyauchi *et al.*

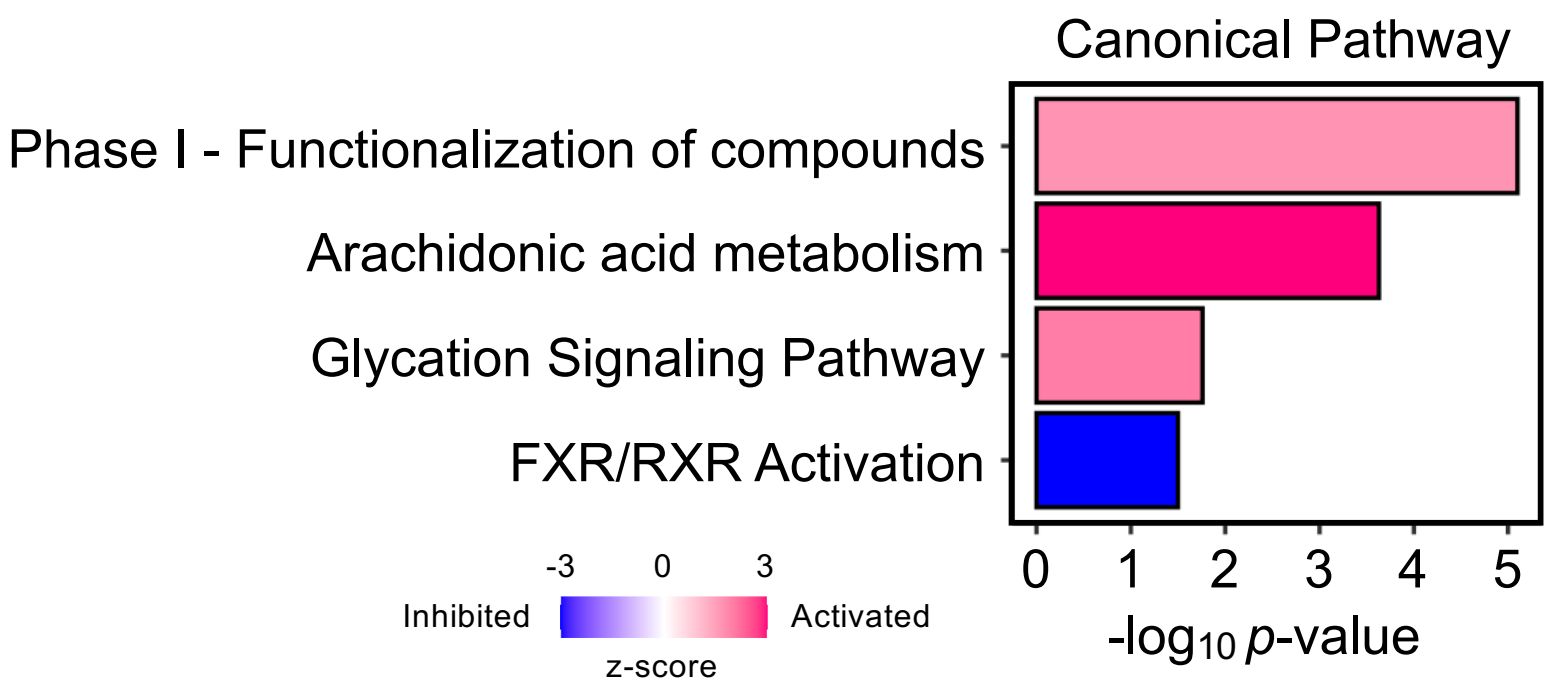

Supplementary Figure 3. Miyauchi *et al.*

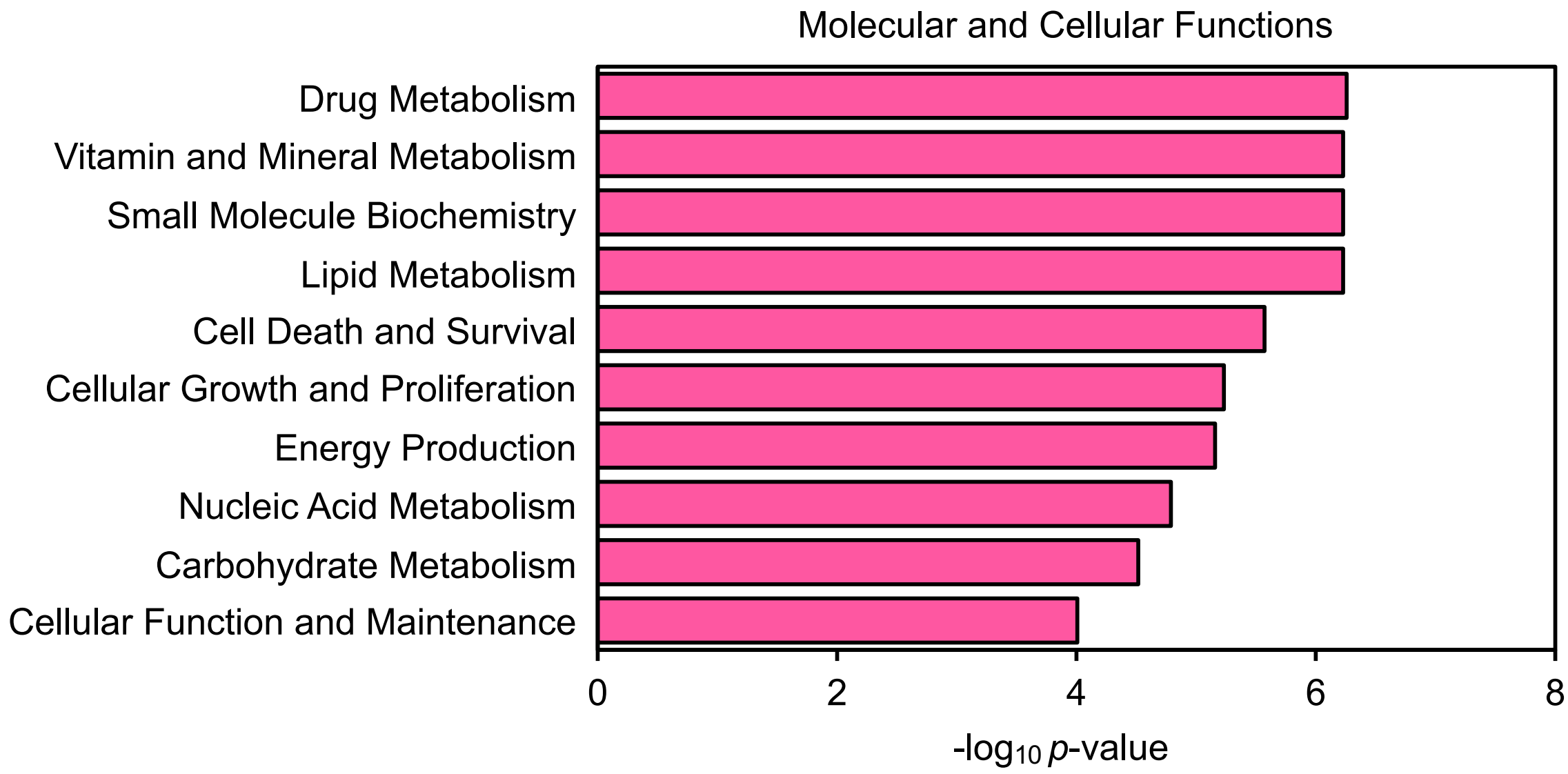

Supplementary Figure 4. Miyauchi *et al.*

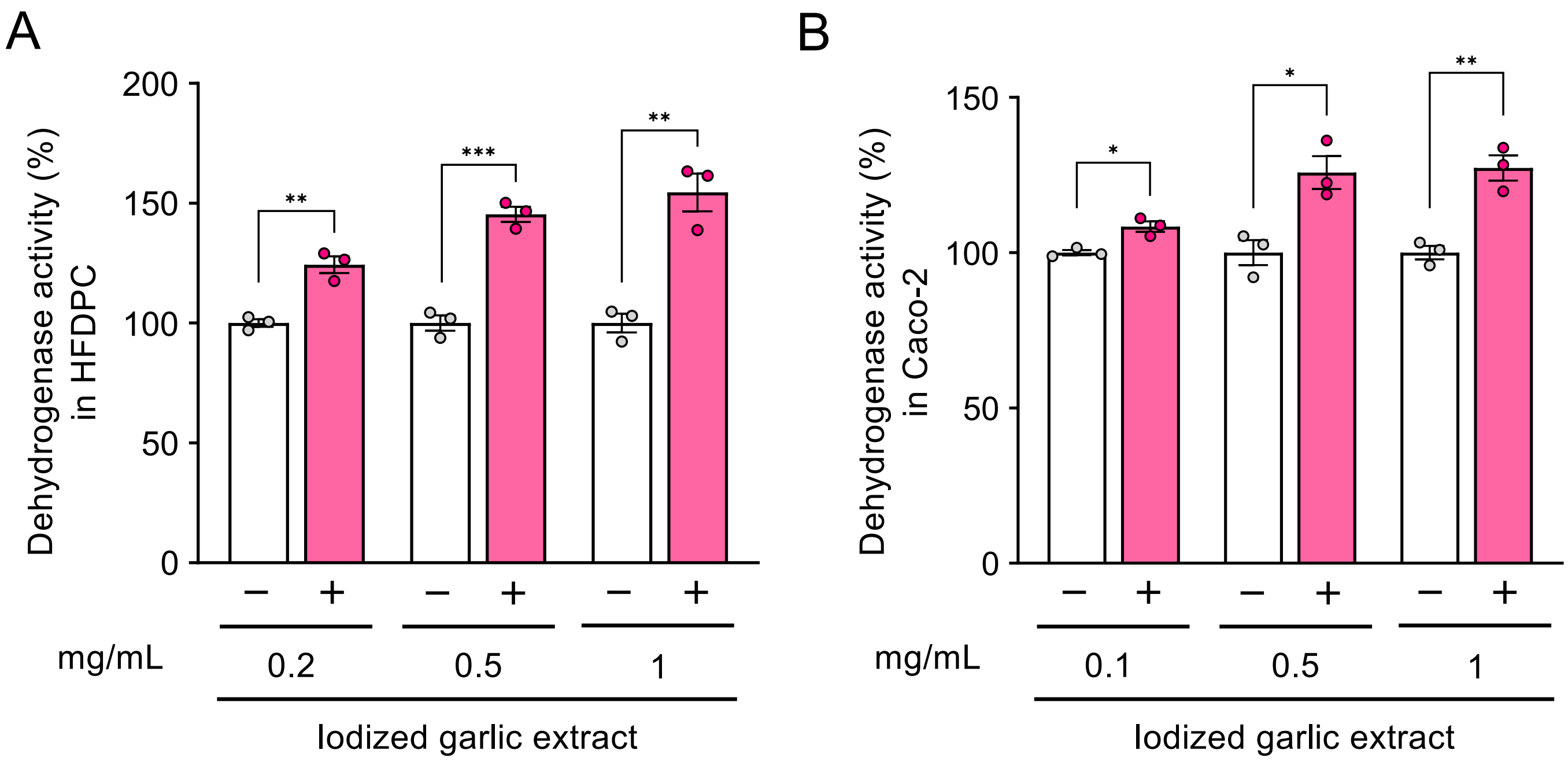

Supplementary Figure 5. Miyauchi *et al.*

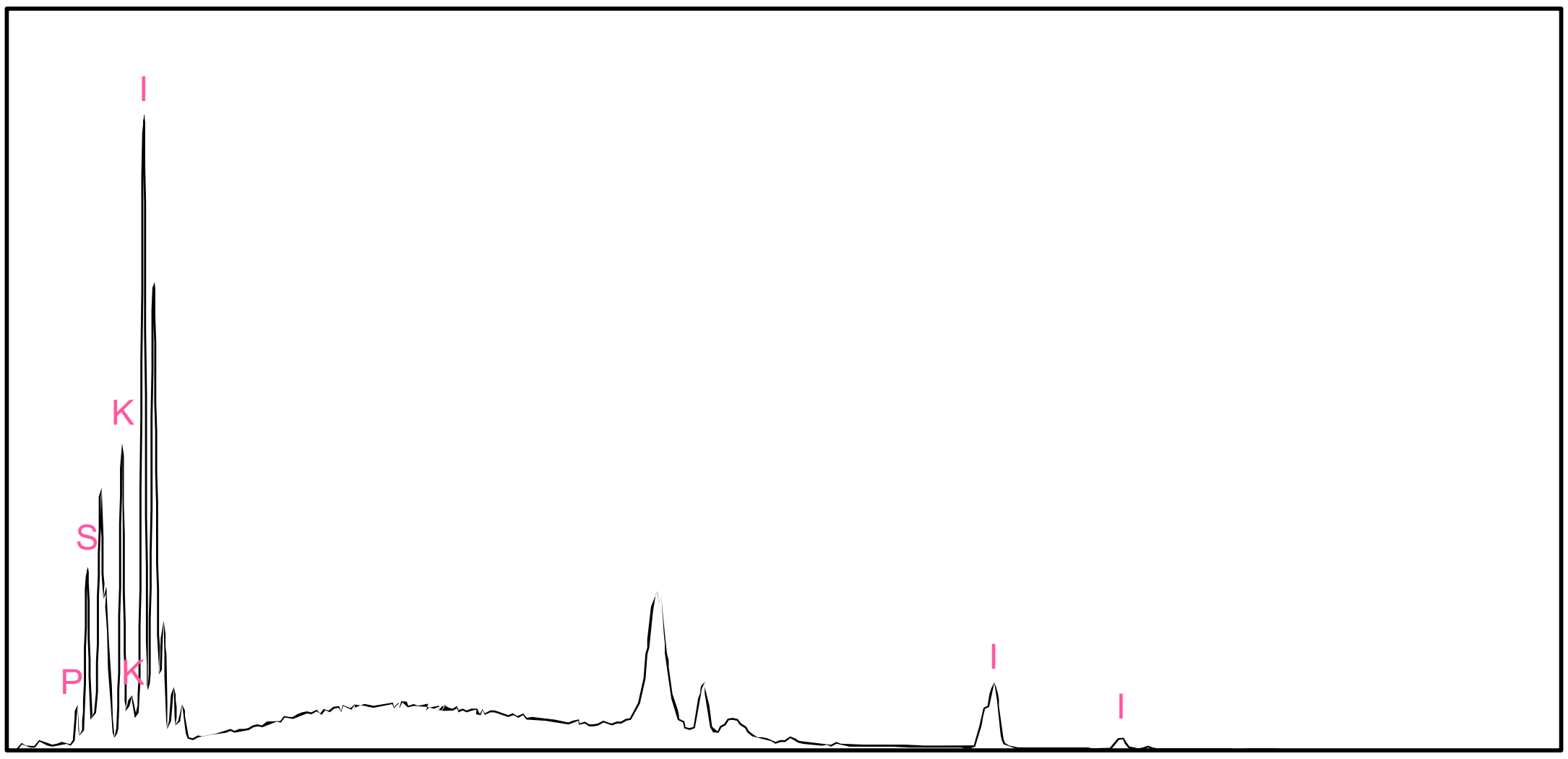

Supplementary Figure 6. Miyauchi *et al.*
