# Supplementary material for: Iodized Garlic Extract Regulates Stress Responses in Epidermal Keratinocytes via the Transcription Factor Nrf2
Source: J Cosmet Dermatol. 2026 Nov 2;25(11):e71228. doi: 10.1111/jocd.71228 (PMC13727960; doi:10.1111/jocd.71228)
Supplement: Supplementary file 2 [file JOCD-25-e71228-s002.docx]

**Supplementary Table 1.**

|  | Forward | Reverse |
| --- | --- | --- |
| *HMOX1* | 5’-TCAGGCAGAGGGTGATAGAAG-3’ | 5’-TTGGTGTCATGGGTCAGC-3’ |
| *AKR1C1* | 5’-GCCGTGGAGAAGTGTAAAGATG-3’ | 5’-CTGGTTGAAGTAAGGATGACATTC-3’ |
| *AKR1C2* | 5’-AACCAGGCCAGTGACAGAAA-3’ | 5’-AGCAGCCTGTGGTTGAAGTT-3’ |
| *AKR1C3* | 5’-CCAATGTCTCTAAAGCCAGG-3’ | 5’-TAGACATCAGGCAAAGCCCT-3’ |
| *DDIT4* | 5’-CTGGACAGCAGCAACAGTG-3’ | 5’-TCACTGAGCAGCTCGAAGTC-3’ |
| *CHAC1* | 5’-CCTGAAGTACCTGAATGTGCGAGA-3’ | 5’-GCAGCAAGTATTCAAGGTTGTGGC-3’ |
| *SESN2* | 5’-TCCGCCACTCAGAGAAGG-3’ | 5’-GGAGGGCGTACAGCAGAG-3’ |

**Supplementary Table 2.**

| (w/w%) | |
| --- | --- |
| Total sugar | 92.8 ± 3.9 |
| Total protein | 3.2± 0.1 |
| Free sugars |  |
| Rhamnose | n.d. |
| Arabinose | n.d. |
| Fructose | 4.5 ± 0.1 |
| Galactose | n.d. |
| Glucose | n.d. |

**Supplementary Table 3.**

| (w/w%) | |
| --- | --- |
| C | 36.9 |
| H | 6.3 |
| N | 1.6 |

**Supplementary Table 4.**

| (mg/100g dry weight) | |
| --- | --- |
| Valine | 3 |
| Proline | 13 |
| Serine | 3 |
| Alanine | 9 |
| Glycine | < 1 |
| Methionine | < 1 |
| Glutamic acid | 6 |
| Aspartic acid | 3 |
| Isoleucine | < 1 |
| Leucine | < 1 |
| Threonine | 3 |
| Tyrosine | < 1 |
| Phenylalanine | < 1 |
| Histidine | 6 |
| Cysteine | 5 |
| Lysine | 27 |
| Arginine | 478 |
